# Towards accelerating model parallelism in distributed deep learning systems

**DOI:** 10.1371/journal.pone.0293338

**Published:** 2023-11-02

**Authors:** Hyeonseong Choi, Byung Hyun Lee, Se Young Chun, Jaehwan Lee

**Affiliations:** 1 Department of Computer Engineering, Korea Aerospace University, Goyang, South Korea; 2 Department of Electrical and Computer Engineering, Seoul, South Korea; 3 INMC & IPAI, Seoul National University, Seoul, South Korea; University of Bologna, ITALY

## Abstract

Modern deep neural networks cannot be often trained on a single GPU due to large model size and large data size. Model parallelism splits a model for multiple GPUs, but making it scalable and seamless is challenging due to different information sharing among GPUs with communication overhead. Specifically, we identify two key issues to make the parallelism being inefficient and inaccurate; an efficient pipelining technique is crucial to maximize GPU utilization and normalizations in deep neural networks may affect the performance due to different statistics sharing of mini-batch. In this work, we address these issues by investigating efficient pipelining for model parallelism and effective normalizations in model / data parallelisms when training a model with large mini-batch in multiple GPUs so that the model performance in accuracy can not be compromised. Firstly, we propose a novel method to search for an optimal micro-batch size considering the number of GPUs and memory size for model parallelism. For efficient pipelining, mini-batch is usually divided into smaller batches (called micro-batch). To maximize the utilization of GPU computing resources, training should be performed with the optimal micro-batch size. Our proposed micro-batch size search algorithm achieved increased image throughput by up to 12% and improved trainable mini-batch size by 25% as compared to the conventional model parallelism method. Secondly, we investigate normalizations in distributed deep learning training for different parallelisms. Our experiments using different normalization methods suggested that the performance with batch normalization can be improved by sharing the batch information among GPUs when performing data parallelism. It was also confirmed that group normalization helped minimizing accuracy degradation when performing model parallelism with pipelining and yielded consistent accuracies for diverse mini-batch sizes.

## 1 Introduction

To achieve high accuracy, it is necessary to use large deep learning models and large datasets are needed to improve the generalization capabilities of the models. Training large-scale models with massive datasets is difficult due to the limited GPU memory size [1–6]. Distributed deep learning using multi-GPU/node can efficiently train large-scale models. Distributed deep learning can be divided into model parallelism and data parallelism according to the parallelization method. When using model parallelism, each worker trains only a part of the model, so that it is possible to train a large-scale model that can not be fit to a single GPU. In data parallelism, multiple workers train the same model with different training data. By using data parallelism, it is possible to quickly train a model with a large dataset using multiple workers. However, recent models with good performance (such as GPT-3 with 175 billion parameters) are near impossible to train on a single GPU. Therefore, models that cannot be trained with a single GPU may dominate the field in the future, and model parallelism, not data parallelism, will be required to train such a large-scale model.

Model parallelism trains a single large-scale deep learning model by dividing it into multiple GPUs. However, making it scalable and seamless is challenging due to different information sharing among GPUs with communication overhead. Specifically, we identify two key issues to make the parallelism being inefficient and inaccurate; pipelining and normalizations.

Firstly, an efficient pipelining technique is crucial to maximize GPU utilization. Since each hidden layer of a deep learning model has a dependency on the output of other hidden layers in model parallelism, each GPU cannot perform the computation until the computation of the other GPU is finished. To alleviate this inefficiency, pipelining technique can be applied to efficiently utilize computational resources [7–9]. When pipelining is applied, training is performed by dividing a mini-batch into smaller micro-batches. Choosing an optimal size of micro-batch is challenging since too small micro-batch will increase communication overhead among GPUs while too large micro-batch will increase the computation time in each GPU. Thus, it is critical to choose an optimal micro-batch size to consider this trade-off to exploit computational resources.

Secondly, effective normalizations in distributed deep neural networks is important to avoid compromising performance due to different mini-batch statistics sharing. Various normalization techniques such as batch normalization and group normalization are important to improve the performance of deep learning model [10, 11]. When performing distributed deep learning in multiple GPUs, however, they may not act as intended. Batch normalization performs normalization using the mean and variance of the input mini-batch. If the size of the input mini-batch is not large enough, the accuracy of the deep learning model can be decreased [11, 12]. This undesirable phenomenon could be related to data parallelism with small mini-batch on multiple workers and model parallelism with pipelining and small micro-batches. Thus, investigating normalizations is important for different parallelism in distributed deep learning.

In this paper, we propose a novel algorithm to determine an optimal micro-batch size so that the pipelining technique can be efficiently applied when performing model parallelism. Our proposed algorithm reduces the overhead of searching micro-batch size by considering the number of GPUs and GPU memory. In addition, we investigate different normalization techniques in a distributed deep learning environment and propose tricks to efficiently train distributed deep learning models with normalizations without deteriorating performance. Here is the summary of the contributions:

- First, we propose a novel micro-batch size search algorithm to determine an optimal micro-batch size for model parallelism with efficient pipelining in multi-GPU environment. Inefficient brute-force search can yield the highest throughput, but with significantly increased overhead. Considering the fact that the largest mini-batch size is the most desirable for a single GPU case, our proposed algorithm starts the search based on both GPU memory size and number of GPUs to reduce the overhead of finding an optimal micro-batch size. Our algorithm was evaluated in our in-house model parallelism implementation with the U-Net, a popular deep neural network for medical imaging. We demonstrate that our algorithm can be used in real-world workloads through experiments in implementing the algorithm and training the U-Net.
- Second, we investigated the effects of normalization on performance degradation in distributed deep learning environments. We observed that batch normalization, a typical choice for deep learning models, degraded accuracy in a distributed environment and we suggested that accuracy degradation can be avoided by sharing the batch information among workers in data parallelism. However, this remedy was not working for model parallelism with pipelining using micro-batches since synchronizing all micro-batches at each batch normalization layer led to training delay. We proposed to use group normalization that did not deteriorate accuracy with small mini-batch or small micro-batch for pipelining.

We trained U-Net, a popular hour-glass shape deep neural network with an encoder and a decoder for medical imaging, in multi-GPUs for the task of CT image enhancement by applying the proposed algorithm. Our model parallel scheme with pipelining increased the image throughput by ∼12% and also increased the size of the trainable mini-batch size by 25%. When performing data parallelism with small mini-batch size, our suggested batch information sharing among workers avoided the issue of accuracy degradation due to batch normalization, a typical choice of normalization in deep learning models. Then, we proposed to use group normalization, which was confirmed in this study to have no difference in accuracy for various mini-batch sizes, for training with micro-batch in our proposed model parallelism with pipelining.

The rest of the paper is organized as follows. In Section 3, we describe how GPUs can be used efficiently when performing model parallelism. We evaluate the performance of the proposed algorithm in Section 5. Also, in Section 4, we describe the problems that may arise from normalization techniques in a distributed deep learning environment. Section 6 shows the performance of normalization techniques in a distributed deep learning environment. We present the related work in Section 2. Finally, we conclude our paper in Section 7.

## 2 Related works

GPipe is a pipeline parallelism library for model parallelization developed by Google [7]. GPipe effectively performs model parallelism by reducing the idle time of GPU by applying the pipelining technique to model parallelism. They have shown experimentally that large-scale models can be trained faster with GPipe without a loss of accuracy. However, they found the optimal micro-batch size for efficient use of GPipe through experiments. The optimal micro-batch size may vary depending on the deep learning model and the mini-batch size. Unlike GPipe, we propose a micro-batch size search algorithm to efficiently apply the pipelining technique.

Xpipe eases GPU idle time caused by processing micro-batches, which is a weakness of Gpipe, through asynchronous pipeline parallelization [9]. They propose a weight prediction technique to find the necessary future weights due to the asynchronous pipeline parallelism. For Inception-V3, XPipe improved throughput by 88.1% on average compared to GPipe in a 4 GPU environment. However, like GPipe, in order to use XPipe efficiently, it is necessary to find the optimal micro-batch size. In this paper, we proposed a micro-batch search algorithm that can efficiently use pipeline parallelism techniques such as XPipe.

When performing model parallelism, resource utilization may be low because training is executed sequentially due to computational dependencies. A. Xu, Z. Huo and H. Huang proposed Layer-wise Staleness and Diversely Stale Parameters (DSP), a training algorithm to solve the problem of inefficient resource utilization [13]. They show that DSP converges well through experiments. In addition, DSP trained models faster than existing algorithms in representative Convolutional Neural Network(CNN) models such as ResNet-50 and ResNet-101. Unlike this paper, we proposed a method to effectively apply the existing pipelining technique.

Batch normalization has a problem that accuracy may be degraded if the size of the mini-batch is not large enough. To solve the problem of accuracy degradation, group normalization has been proposed [11]. Unlike Batch Normalization, which performs normalization at the batch dimension, group normalization processes image features by dividing them into groups like existing image processing techniques such as HOG and SIFT. Therefore, even if the size of the mini-batch is small, it shows high accuracy. However, in this paper, experiments were performed only on multi-GPU-based data parallelization. We have shown through experiments that group normalization works well even when multi-GPU-based model parallelization is performed.

The Large Mini-Batch Object Detector (MegDet) performs training faster than the existing system by using multiple GPUs to increase the large mini-batch size [12]. MegDet proposed a learning rate warmup strategy and Cross-GPU Batch Normalization (CGBN) to achieve high accuracy even when the large mini-batch size is increased. Similar to our experimental results, their paper also shows that when data parallelization is performed using multiple GPUs, accuracy can be improved by sharing a mini-batch between GPUs. However, unlike our work, MegDet has a limitation in that it only conducted research on data parallelization.

## 3 How to use GPU more efficiently when performing model parallelism

### 3.1 Model parallelism with pipelining

If training with a single GPU is not possible due to the large size of the training model, the model is divided and trained on multiple GPUs through model parallelism. There are three typical methods of dividing a model across multiple GPUs—layer division, feature division, and hybrid division [8, 14–16]. In layer division, one or more layers are stored in each GPU to perform training. In the case of layer division, the feature map data calculated on the GPU_*n*_ is transferred to the GPU_*n*+1_ during feed-forward. Conversely, when performing back propagation, the gradient which calculated by the loss calculated on the GPU_*n*_ is transferred to the GPU_*n*−1_. When deep learning is performed in the feature division method, each GPU generates features using model parameters and input features. Then, each GPU passes the generated features to the other GPUs. Hybrid division is a combination of layer division and feature division. Hybrid division can be applied when the deep learning model is very large. When performing model parallelism, each GPU is dependent on the output of other GPUs due to the modularity of deep learning. Accordingly, each GPU is placed in an idle state until it receives the feature map data generated by the other GPU. In order to efficiently use the computing resources of GPUs when performing model parallelism, the idle time of each GPU should be reduced by optimizing communication between GPUs.

**Fig 1:**
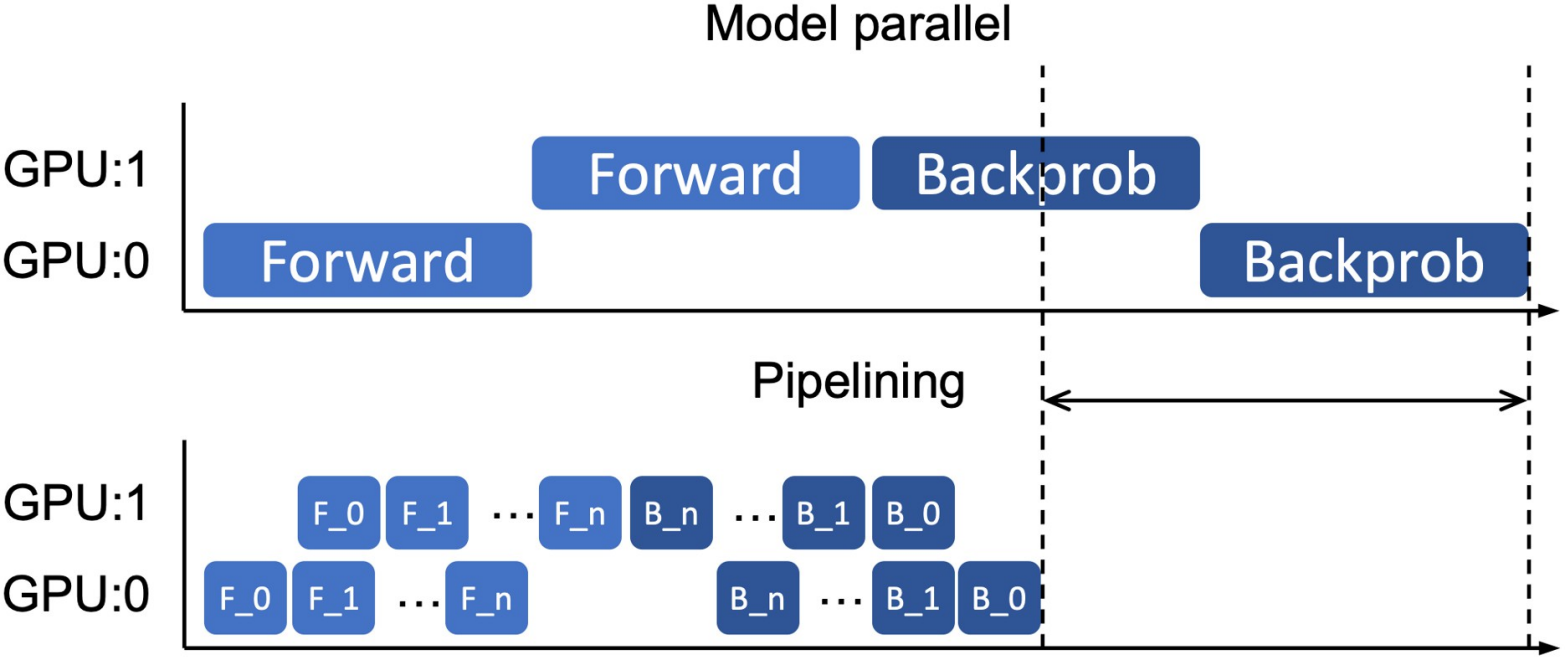
Reduction of training time by applying pipelining technique.

As described earlier, due to the characteristics of deep learning models, when model parallelism is applied, the computing resources of all GPUs used cannot be fully utilized. To solve this problem, the pipelining technique can be applied. When pipelining is applied to model parallelism, training is performed by dividing the training mini-batch into smaller mini-batches. A small-sized mini-batch divided into a mini-batch for pipelining is called a micro-batch. The top of Fig 1 shows the timeline when performing the existing model parallelism technique, and the bottom shows the timeline when pipelining is applied. As shown in the Fig 1, applying a pipelining reduces the idle time of each GPU, so that training can be performed faster.

### 3.2 Efficient model parallelism architecture

When performing model parallelism, the feature map data generated as a result of feed forwarding from the GPU_*n*_ is transferred to the GPU_*n*+1_. The GPU_*n*+1_ performs feed forwarding using the feature map data received from the nth GPU and the model parameters it has. When performing general model parallelism, the input mini-batch is trained as a single matrix computation. Therefore, in the general model parallelism technique, the GPU_*n*+1_ is placed in an idle state until the GPU_*n*_ completes feed forwarding. Similarly, when backpropagation is performed, the GPU_*n*−1_ is idle until the backpropagation is completed on the GPU_*n*_ and the gradients calculated by the GPU_*n*_ is transferred to the GPU_*n*−1_. In this paper, we apply the pipelining technique to solve the problem of GPU utilization, which is a problem of existing model parallelism technique.

**Fig 2:**
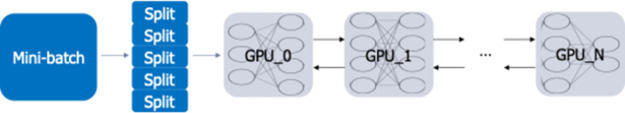
Model parallelism with pipelining technique.

Fig 2 shows the model parallelism architecture to which the pipelining technique is applied to efficiently use the computing resources of the GPU. The order of operation of the model parallelism architecture in the Figure is as follows:

- **1**. Read mini-batch from storage into CPU memory.
- **2**. Split the input mini-batch into micro-batches and send a micro-batch to the GPU which has an input layer
- **3**. The GPU_*i*_ performs feed forwarding using the input micro-batch and model parameters.
- **4**. The GPU_*i*_ sends the feature map data, which is the result of feed forwarding, to the GPU_*i*+1_.
- **5**. After feed forwarding is completed for all micro-batch, backpropagation is performed for each micro-batch in the reverse order of feed forwarding.
- **6**. Update model parameters after performing backpropagation for all micro-batches.

**Fig 3:**
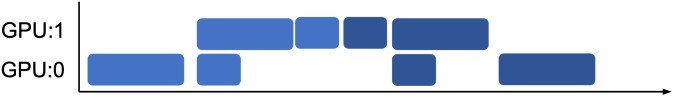
Training when the size of the micro-batch is too large.

**Fig 4:**
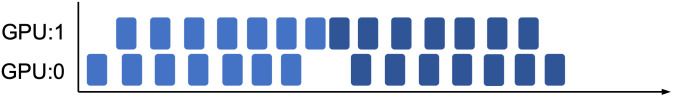
Training when the size of the micro-batch is too small.

In the above way, the computing resources of the GPU can be used efficiently. However, if the input mini-batch is not divided into micro-batches of an appropriate size, the computing resources of the GPU cannot be used efficiently. Therefore, in order to improve the performance by applying the pipelining technique, it is important to find a micro-batch of an appropriate size. Fig 3 shows the training timeline when the size of the micro-batch is too large. As shown in Fig 3, if the size of the micro-batch is similar to that of the mini-batch, the idle time of the GPU is long, so computing resources cannot be used efficiently. Fig 4 shows the training timeline when the micro-batch size is too small. If the micro-batch size is too small, the GPU’s computing resources cannot be used efficiently due to increased communication between GPUs.

### 3.3 Proposed micro-batch search algorithm

**Algorithm 1** Search micro-batch size

1: *S*_−1_ ← 0

2: *S*_0_ ← 0

3: **if** *s* ≥ *mini batch* **then**

4:  $S_{1}\leftarrow \frac{mini\;batch}{numGPUs}$

5: **else**

6:  *S*_1_ ← *s*

7: *n* ← 0

8: *t*_0_ ← *training time with S*_0_

9: *t*_1_ ← *training time with S*_1_

10: **while** 0 ≤ *t*_*n*_ − *t*_*n*+1_ ≤ *T* **do**

11:  $S_{n+1}\leftarrow \frac{S_{n}+S_{n-1}}{2}$

12:  *t*_*n*+1_ ← *training time with S*_*n*+1_

13:  *n* ← *n* + 1

14: **return** *S*_*n*_

Homogeneous training environment refers to an environment in which all workers have the same GPU performance and memory size. In homogeneous training environment, as the number of GPUs used increases, the training mini-batch size increases. As the training mini-batch size increases, the search for an optimal micro-batch size may become difficult as the scope of the search increases. In this section, we describe a micro-batch size search algorithm to efficiently apply pipelining. The algorithm is a pseudo-code of the micro-batch size search algorithm proposed in this paper. The Algorithm 1 is a micro-batch size search algorithm proposed in this paper. To reduce the overhead caused by the micro-batch size search, we initialize the micro-batch size based on the number of GPUs used and the GPU memory size and then start the micro-batch search. The larger the batch size input to the GPU, the more computational resources of the GPU can be utilized. Therefore, if the memory required to train the input mini-batch is larger than the size of the GPU memory, the initial micro-batch size is initialized to the maximum mini-batch size that can be trained in the GPU memory. However, when the size of the input mini-batch is smaller than the size of the mini-batch that can be trained on a single GPU, if the initial micro-batch size is the same as the mini-batch, the micro-batch training becomes the same as the mini-batch training. So, if the input mini-batch is smaller than the mini-batch size that can be trained on a single GPU memory, the initial micro-batch size is initialized as the size of the input mini-batch divided by the number of GPUs.

In Algorithm 1, *s* is the largest mini-batch size that can be trained with a single GPU. *mini batch* is the size of the input mini-batch and *numGPUs* is the number of GPUs used. *S*_0_ is 0, the micro-batch size before initialization. *S*_1_ is the micro-batch size obtained by the initialization scheme described above. When the *n*^*th*^ size is *S*_*n*_, the size of the *n* + 1^*th*^ micro-batch *S*_*n*+ 1_ is $\frac{S_{n}+S_{n-1}}{2}$. *t*_*n*_ is the average time trained for 10 iterations with the micro-batch size *S*_*n*_. We compare *t*_*n*+ 1_, which is the time when the model is trained with the size of the micro-batch *S*_*n*+1_, and *t*_*n*_, which is the time when the model is trained with the size of the micro-batch *S*_*n*_. *T* is the threshold for stopping the micro-batch size search. If the difference in training time (*t*_*n*_ − *t*_*n*+1_) trained with micro batches of two different sizes (*S*_*n*_ and *S*_*n*+1_) is less than *T*, the search is stopped. Also, the search stops even if *t*_*n*+1_ is greater than *t*_*n*_. In our experiments, we set the threshold *T* to 0.01 s.

## 4 Normalization methods in distributed deep learning

There are two main normalization techniques, the first is whitening and the second is batch normalization. First, whitening [17] is a technique of whitening inputs to quickly converge the network when performing learning. Whitening transforms the inputs to mean 0, variance 1, and makes them unrelated. However, whitening has a large amount of computation. Also, whitening proceeds independently of backpropagation, so only some parameters may become large. Therefore, recent deep learning models use the normalization technique to speed up training by removing the internal covariate shift. Second, batch normalization [10] performs normalization using the mean and variance of the inputs of each layer, thereby removing the internal covariate shift [10]. Batch normalization uses mini-batch for normalization. A mini-batch that is not large enough is difficult to represent the entire data set. Therefore, when using the batch normalization technique, if a mini-batch of sufficient size is not used, the accuracy of the deep learning model decreases.

For large-scale deep learning models, it is difficult to achieve high accuracy through batch normalization because the size of the mini-batch cannot be large enough due to GPU memory limitations. Distributed deep learning can be performed to increase the size of the input mini-batch. The representative techniques of distributed deep learning are data parallelism and model parallelism. Data parallelism increases the size of the overall input mini-batch because multiple workers train the same training model with different training data, but the size of the mini-batch each worker trains does not increase. Therefore, the size of the input mini-batch of the batch normalization layer of the model trained by each worker does not increase either. So, data parallelism reduces accuracy because the size of the input mini-batch that each worker trains is small. In order to increase the accuracy of the model, the workers share each other’s input mini-batch information through communication to increase the size of the input mini-batch of the batch normalization layer [12].

Model parallelism can increase the size of the input mini-batch, as each worker trains part of one model with the same input data. Therefore, in the case of general model parallelism, the accuracy can be improved through batch normalization. However, if the pipelining technique is applied to speed up training, training is performed by dividing the input mini-batch into smaller micro-batches. Therefore, there is a disadvantage in that the size of the input becomes small and the accuracy decreases. To solve the problem of batch normalization, the decrease in accuracy due to the size of a small input mini-batch, group normalization was proposed. Group normalization [11] is designed based on the existing image processing techniques such as Histogram of Oriented Gradient(HOG) [18] and Scale Invariant Feature Transform(SIFT) [19], which divides image features into groups and processes them. Therefore, unlike batch normalization, group normalization divides the input features of each layer into several groups and performs normalization for each group, so that it is not affected by the size of the input mini-batch.

## 5 Performance evaluation of proposed algorithm

To evaluate the performance of the micro-batch size search algorithm proposed in this paper, we implemented the algorithm using Python 3.7.6. We evaluated the performance of the proposed algorithm in the following experimental environment. We used Pytorch 1.4.0, a deep learning framework, and CUDA 10.0, and NVIDIA driver 418.56 to use GPU. In addition, two NVIDIA GeForce GTX 1080 Ti were used as GPUs for performing deep learning. U-Net [20] has a U-shaped network structure with a structure that combines a contracting path and an expanding path. U-Net has a problem in that the amount of communication between the contracting path and the expanding path is large. U-Net was used as the training model, and training was performed by dividing the contracting path and expanding the path to each GPU. The reason we selected U-Net as an evaluation target is because it is a representative semantic segmentation model. Since semantic segmentation models are constructed based on the existing image classification model, U-Net can represent most models.

In PyTorch, part of the model can be located on different devices using the to(device) API. PyTorch launches CUDA operations asynchronously. We partitioned the model using the to(device) API. Also, due to the characteristic of PyTorch, which launches CUDA operations asynchronously, a pipeline can be implemented by inputting micro batches without the need to create multiple threads.

**Fig 5:**
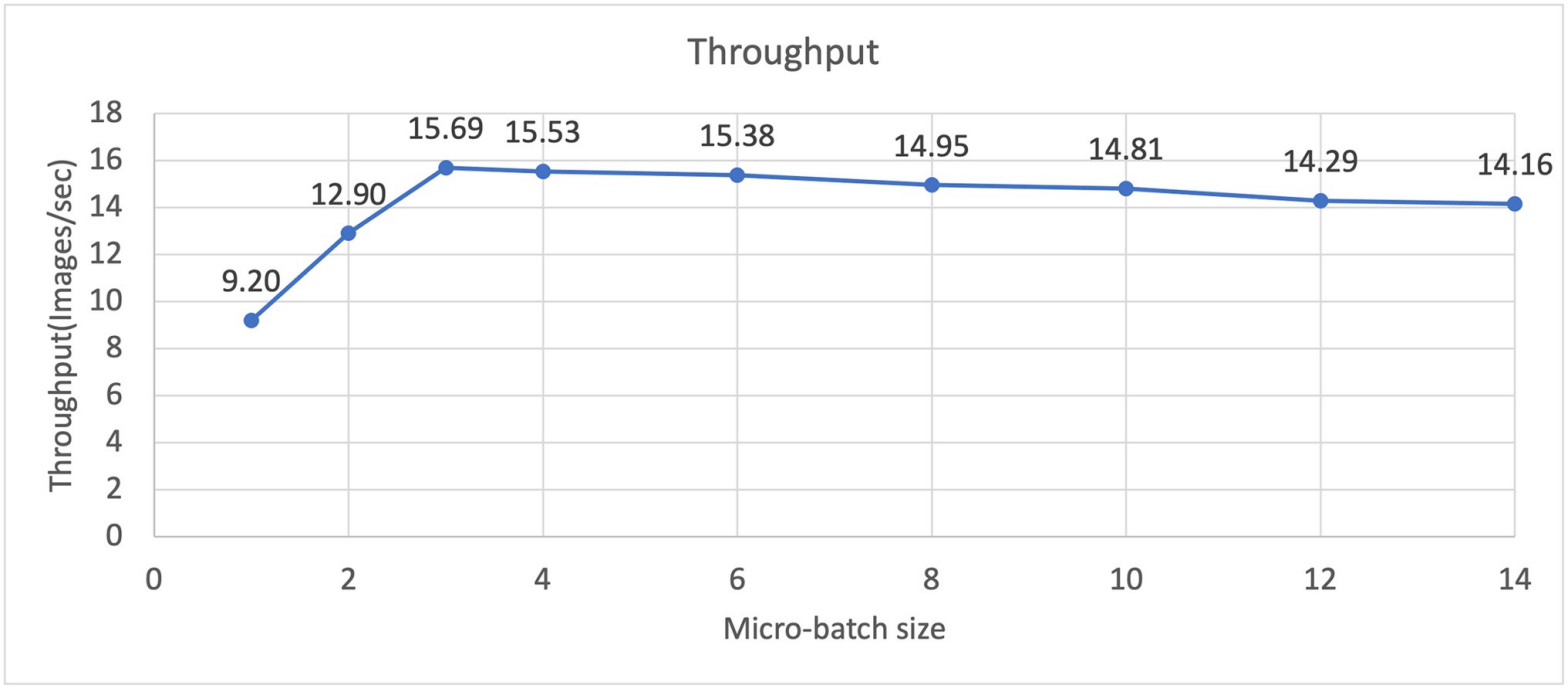
Graph of throughput change with an increasing micro-batch size when mini-batch size is 16.

**Fig 6:**
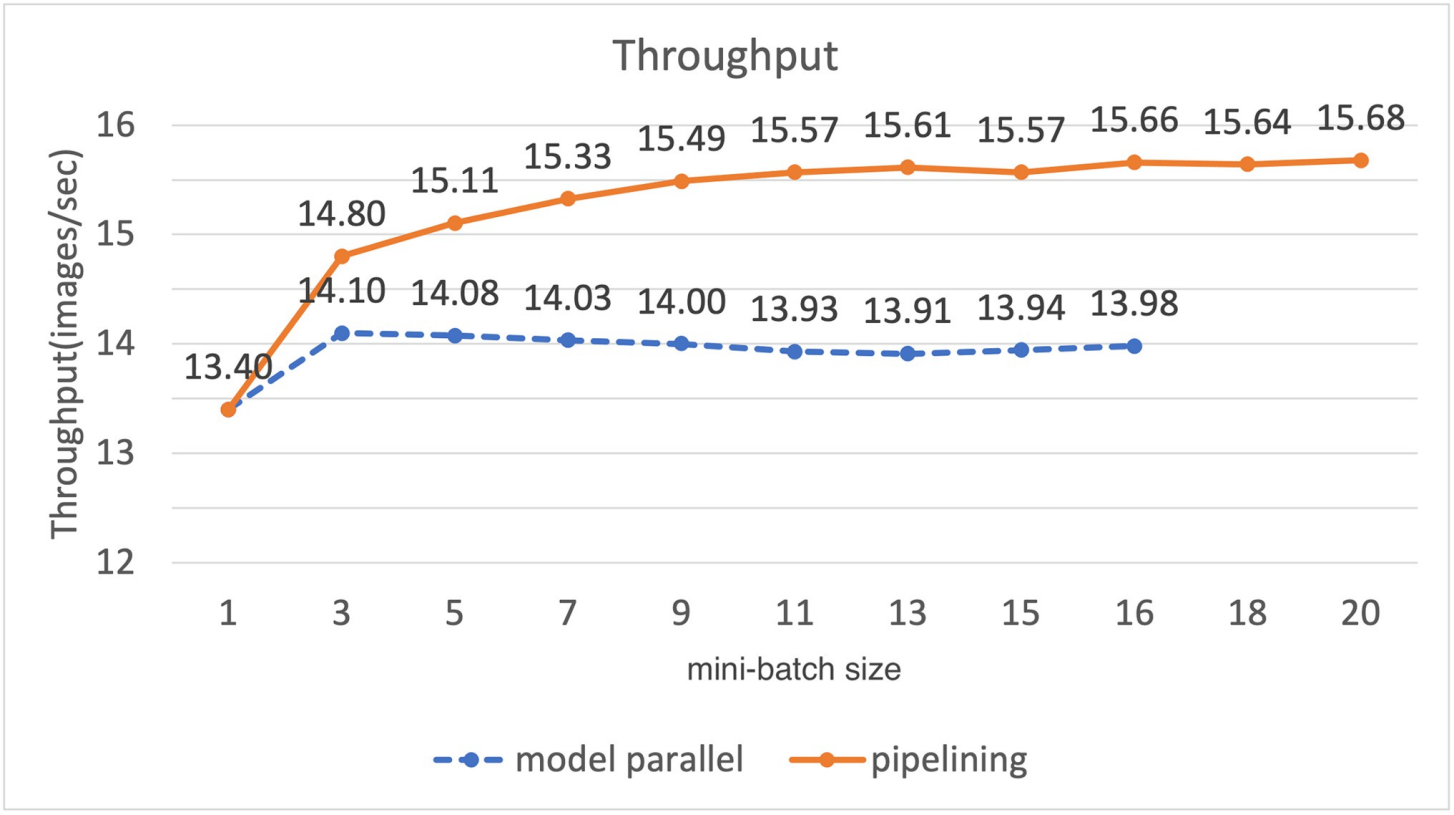
Graph of throughput change with a mini-batch size.

Before evaluating the performance of the algorithm proposed in this paper, we checked the learning performance by the micro-batch size. To justify our argument that finding the optimal micro-batch size can reduce execution time, the image throughput per second was measured for each micro-batch size. We can simply calculate the execution time per image by inverse calculation of the image throughput per second. Fig 5 is a graph showing the change in image throughput per second with the increase in micro-batch size when the training mini-batch size is 16. As shown in the Fig 5, when the micro-batch size was increased from 1 to 3, the image throughput increased by about 70% from 9.2 images/sec to 15.69 images/sec. However, if the micro-batch size is larger than 3, the throughput is reduced compared to when the micro-batch size is 3. When the micro-batch size is 14, the image throughput is reduced to 14.16 images/sec, and the image throughput is reduced by about 11% compared to when the micro-batch size is 3. The reason why this experiment shows the above results is as follows. If the micro-batch is too small, training on a large number of micro-batches is required to train one mini-batch. Therefore, the number of times that the result computed by each GPU needs to be transferred to another GPU increases. As a result, communication between GPUs increases compared to the computation of GPUs, so that the computational resources of the GPU cannot be efficiently utilized. If the micro-batch is larger than the appropriate size, the micro-batch size approaches that of the mini-batch. Therefore, the computation time of each GPU becomes longer, and the idle time of the GPU that does not perform computation also becomes longer. In order to efficiently use the GPU to perform deep learning quickly, it is necessary to find the optimal micro-batch size. Fig 6 shows the image throughput of vanilla model parallelism and model parallelism with pipelining. When performing model parallelism by applying pipelining, we use the micro-batch size calculated through the search algorithm 1 proposed in this paper. We conducted experiments by increasing the size of the mini-batch to the maximum size that can be trained. From the graph in the figure, model parallelism by applying pipelining shows a higher image throughput for all mini-batch sizes than when pipelining is not applied. When the mini-batch size was 16, the image throughput for model parallelism with pipelining was 15.66 images/sec, and for model parallelism without pipelining, the image throughput was 13.91 images/sec. By training to the micro-batch size, image throughput increased by up to about 12%. When pipelining is applied, it is possible to increase the size of the trainable mini-batch as well as the image throughput compared to the existing model parallelism. With GeForce GTX 1080 Ti, the maximum trainable mini-batch size when training U-Net is 10. When we perform model parallelism using two GeForce GTX 1080 Tis, the size of the trainable mini-batch increased by 60%, and we were able to train up to 16 elements in the mini-batches. When performing learning by applying pipelining, the unit for deep learning on the GPU is not a mini-batch, but a micro-batch that is smaller than a mini-batch. Therefore, the memory required to perform deep learning operations is less than when training mini-batch. Therefore, if pipelining is applied, the model can be trained with a larger mini-batch than model parallelism without pipelining, since the memory is used in a different way. Through this experiment, we have shown that image throughput can be increased by up to 12% compared to basic model parallelism when training is performed with the micro-batch of the size found through the proposed micro-batch size search algorithm. Furthermore, we show that by applying pipelining to the model parallelization technique, not only the image throughput but also the trainable mini-batch size can be increased compared to the conventional model parallelism. If pipelining is applied to model parallelism, it is possible to train a mini-batch twice as much as when deep learning is performed using a single GPU.

## 6 Performance of normalization methods in distributed deep learning

To evaluate the performance of normalization in distributed deep learning, we constructed a distributed deep learning environment using 4 NVIDIA TITAN Xp units. We first trained U-Net with the same data and optimizer on a single GPU under fixed random seed until 80 epochs for reproduction. Then we restarted training the model under various parallelism methods after 80 epochs. After 80 epochs, we doubled the mini-batch size every 20 epochs. Also, we trained the U-Net by halving the learning rate every 20 epochs from 110 epochs. When a deep learning model is trained using the model parallelism technique without pipelining, it shows the same results as training with a single GPU. Therefore, we evaluate the performance of normalization in a distributed deep learning environment based on the performance when U-Net is trained with a model parallelization technique without pipelining. In all experiments, we input data into the model in the same order. We use Peak Signal-to-Noise Ratio (PSNR), which can evaluate the loss information of the generated image for performance evaluation.

### 6.1 Performance of batch normalization in distributed deep learning

**Fig 7:**
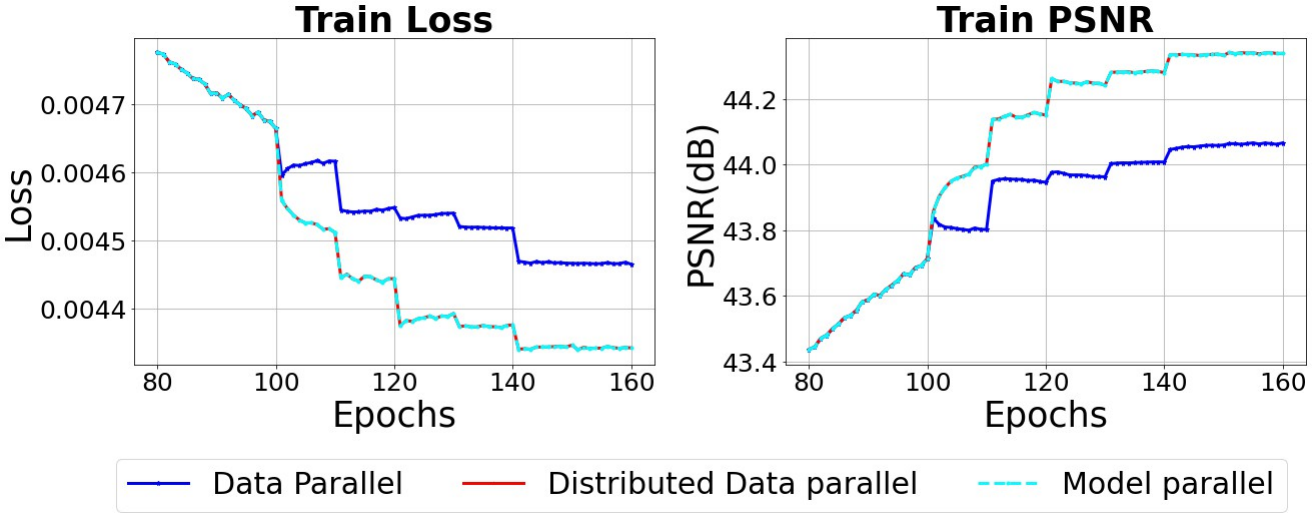
Training performance when batch normalization is applied in a distributed deep learning environment.

**Fig 8:**
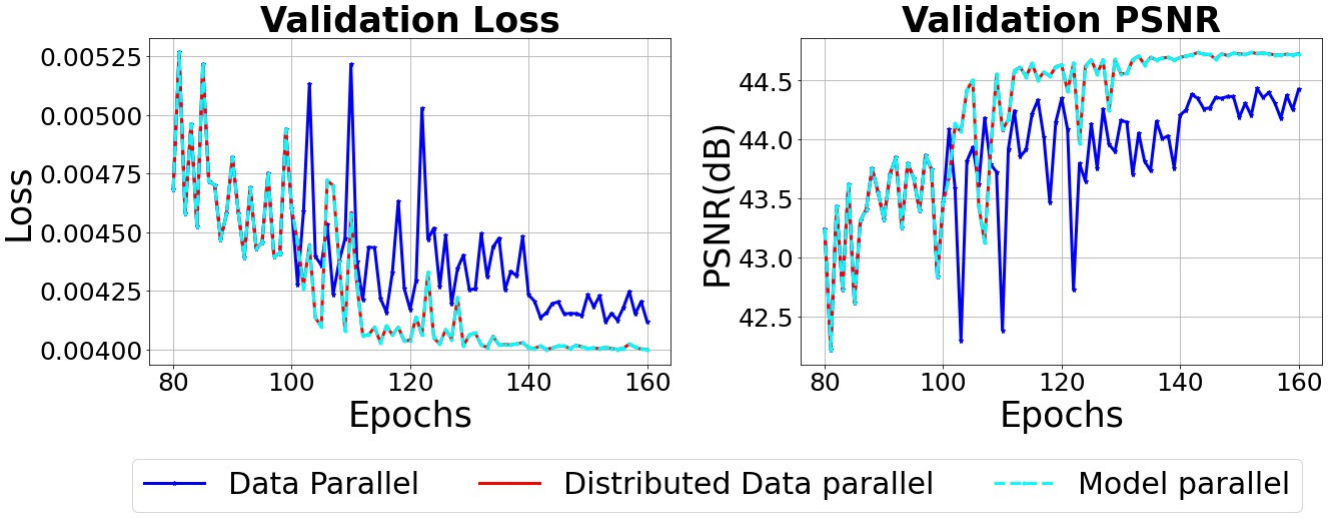
Validation performance when batch normalization is applied in a distributed deep learning environment.

Figs 7 and 8 show that the performance of deep learning is degraded due to batch normalization when performing distributed deep learning with a data parallel technique. Looking at Fig 7, we can see that the training loss that decreases after increasing the mini-batch is smaller than the model parallel when the data parallel technique is used. At 100 and 120 epochs where the size of the mini-batch is doubled, the training loss of the model parallel decreases significantly. Also, the graph shows that the decrease in the training loss of data parallel at 100 and 120 epochs where the mini-batch is doubled is smaller than that of model parallel. However, we found that the reduction in loss at 140 epochs was almost similar to data parallel and model parallel. The reason for this result at 140 epochs seems to be that in the case of model parallel, the training loss has already sufficiently converged. Learning rate decay showed the same effect in both data parallel and model parallel cases. In the case of training PSNR, the performance of the data parallel was lower than the performance of the model parallel as in the training loss. The learning rate decay showed the same effect in both data parallel and model parallel in training PSNR. Fig 8 shows the validation loss and validation PSNR of data parallel and model parallel. Looking at the Fig 8, data parallel is generally lower than model parallel after 100 epochs for both validation loss and validation PSNR. Also, the validation loss of model parallel decreases more stably compared to data parallel. Similarly to the validation loss, the validation PSNR increases more stably in model parallel than in data parallel.

Figs 7 and 8 show that batch normalization can be effectively applied by sharing mini-batches through communication between workers when performing data parallel. Looking at Fig 7, the training loss of data parallel decreases in the same way as the model parallel. In Fig 7, the training PSNR also increases in the same way as data parallel and model parallel. In Fig 8, validation performance is also improved by sharing mini-batch between workers like training performance. The validation loss and validation PSNR of model parallel and validation loss and validation PSNR of data parallel are the same. Therefore, when training a model through data parallel, information about the mini-batch must be shared through communication between workers to effectively utilize batch normalization.

**Fig 9:**
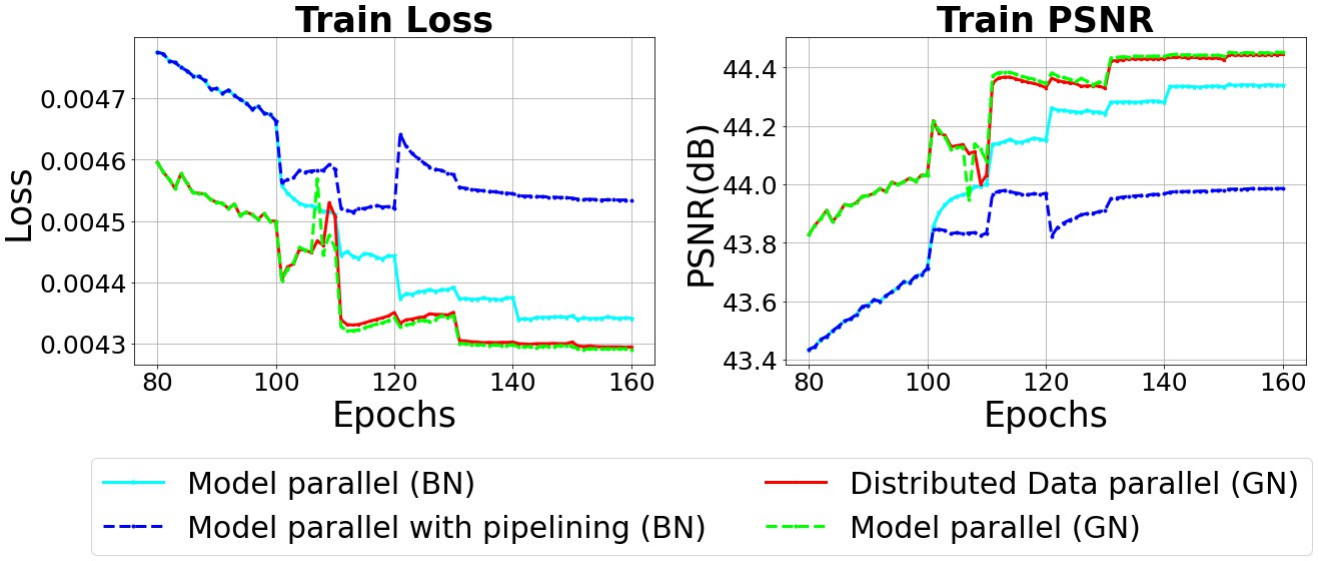
Training performance when batch normalization and group normalization are applied in a distributed deep learning environment.

**Fig 10:**
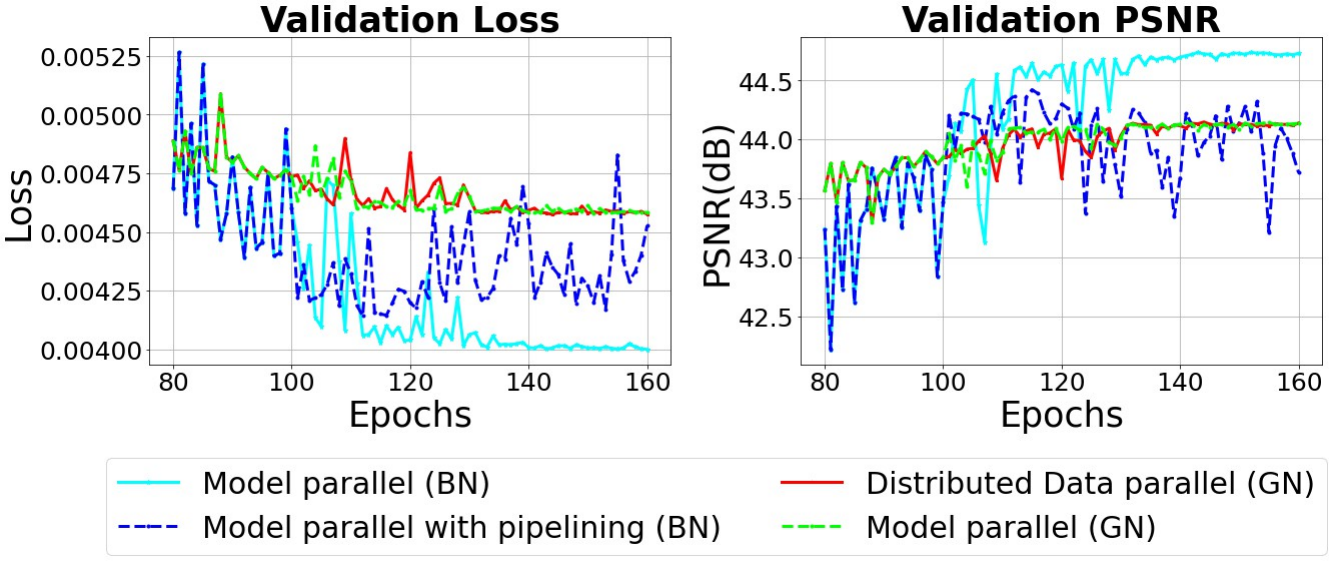
Validation performance when batch normalization and group normalization are applied in a distributed deep learning environment.

In Figs 9 and 10, model parallel is the result of training the model using the general model parallelism technique, and model parallel with pipelining is the result of applying pipelining to the general model parallelism technique. As shown in the Fig 9, since training is performed with the mini-batch size as 1 until 100 epochs, there is no difference in train loss and train PSNR between pipelining and non-pipelining. However, after 100 epochs, when training is performed with the training mini-batch size set to 2 and the micro-batch size to 1, the train loss with pipelining is higher and the train PSNR is lower with pipelining than without pipelining.

In addition, when training was performed by increasing the training mini-batch to 4 and micro-batch to 2 at 120 epochs, applying pipeling increased the train loss and decreased the train PSNR than before 120 epochs. At 140 epochs, when the size of the training mini-batch was doubled and the micro-batch was increased to 3, the train loss and train PSNR were significantly changed when pipelining was not applied. However, when pipelining was applied, the changes in train loss and train PSNR were very small compared to those without pipelining.

In Fig 10, when training is performed with general model parallelism, the validation loss and validation PSNR converge as training is performed. However, when pipelining is applied, we can see that the loss and PSNR do not converge because the model is not trained after 100 epochs. Therefore, the results of this experiment show that when pipelining is applied, a micro-batch smaller than a mini-batch is input, which can reduce the performance of batch normalization and reduce the accuracy of the model.

### 6.2 Performance of group normalization in distributed deep learning

Figs 9 and 10 show the results of performing the experiment by changing the batch normalization layers of U-Net used in the previous experiment to group normalization layers. Looking at Fig 9, the training performance of the model parallel is better when group normalization is applied. When the learning rate is reduced from 0.01 to 0.005 at 110 epochs, it can be seen that the model parallel converges the loss and PSNR faster than the data parallel. After 110 epochs, the model trained in model parallel shows better performance than data parallel. Unlike batch normalization, there is little difference between data parallel and model parallel in the reduction of train loss and increase in train PSNR after changing the size of the training mini-batch.

Fig 10 shows validation performance when group normalization is applied instead of batch normalization. Looking at the Fig 10, it can be seen that model parallel trains the model more stably compared to data parallel. However, the performance difference between model parallel and data parallel is smaller in group normalization than in batch normalization. Also, in the case of batch normalization, the model trained in data parallel shows lower performance than the model trained in model parallel because the loss and PSNR do not converge sufficiently at 160 epochs. On the other hand, in the case of group normalization, the loss and PSNR of the model trained with the data parallel method converge sufficiently, so there is no difference in performance from the model parallel. Unlike batch normalization, group normalization does not cause any loss in model performance due to the small size of the training mini-batch.

## 7 Conclusion

In this paper, we proposed a micro-batch search algorithm to efficiently apply pipelining when performing model parallelism. According to the results of our experiments, image throughput improved by up to about 12% compared to model parallelism without pipelining when training with micro-batches of a size calculated using the proposed algorithm was performed. In addition, we confirmed through experiments the problems that may arise due to normalization when performing distributed deep learning and how to solve them. When performing data parallelism, the performance of batch normalization can be improved by sharing mini-batches between GPUs. By using group normalization rather than batch normalization, it is possible to prevent the decrease in accuracy when performing model parallelism to which the pipelining technique is applied.

So, we showed the problems that can arise from using multiple GPUs when performing data parallelization as well as model parallelism, and showed that these problems can be solved by applying previous studies. In addition, existing studies such as Gpipe have suggested ways to optimize memory and computation so that training can be performed efficiently. However, unlike previous studies, we proposed a method to find a micro-batch that can minimize the GPU idle time that can occur by performing model parallelism.

In the future, we plan to conduct experiments using more diverse deep learning models and data sets and analyze performance in various environments such as asynchronous model parallelism(e.g., AMPNet) using more GPUs. We also plan to develop more sophisticated micro-batch search algorithms as a future goal.
